# Draft genome sequence of *Salinicoccus* sp. JC660 isolated from a wastewater treatment plant

**DOI:** 10.1128/mra.00754-24

**Published:** 2025-06-18

**Authors:** Chintalapati Sasikala, Lakshmanan Vighnesh, Chintalapati Venkata Ramana

**Affiliations:** 1 Smart Microbiological Services, Secunderabad, India; 2 Department of Plant Sciences, School of Life Sciences, University of Hyderabad 98850, Hyderabad, India; University of Southern California, Los Angeles, California, USA

**Keywords:** *Salinicoccus*, draft genome, nutrient recovery

## Abstract

Here, we report the draft genome sequence of a salmon rose bacterium, *Salinicoccus* sp. JC660, isolated from a wastewater treatment plant. The genome assembly has a GC content of 48.5% and a length of 2,686,240 bp.

## ANNOUNCEMENT

Strains of the genus *Salinicoccus* are Gram-stain-positive, non-motile, non-spore-forming cocci. MK-6 is the predominant respiratory quinone and has a DNA G + C content of 46–51 mol% (1). The genus *Salinicoccus* comprises 18 species with validly published names (https://lpsn.dsmz.de/genus/salinicoccus), which were isolated from diverse environmental and food sources, including soda lake, saline soil, and fermented seafood (2). The draft genome of *Salinicoccus* sp., isolated from a treatment plant for waste discharge after *Lactobacillus* fermentation, is reported here.

Strain JC660 was isolated from a waste discharge sample collected from an industry located in Jeedimetla, Hyderabad, India (Global Positioning System coordinates of sampling site: 22°98′ N 71°47′ E) by plating 1 mL of the sample on nutrient agar (Nutrient agar; Himedia M001), followed by incubation and cultivation according to Ramana et al. (1). A single colony was used for genomic DNA extraction using the NucleoSpin Microbial DNA extraction kit. Library preparation and sequencing were outsourced to the BGI group (GCM 10K type strain sequencing project), China. The library was prepared using the Agencourt AMPure XP-Medium kit. The paired-end sequencing of (150 × 2 bp) fragment library by Illumina Hiseq X Ten resulted in about 3,848,599 paired-end raw reads, which were later quality trimmed using Trimmomatic version 0.36 (3), assembled using SOAPdenovo version 1.05 software (4), and annotated using NCBI Prokaryotic genome annotation pipeline (PGAP) (5).

The 16S rRNA gene sequence of strain JC660 showed 100% identity with *Salinicoccus halitifaciens* JC90^T^ (HE578181), followed by *Salinicoccus kekensis* DSM 23173^T^ (OBQF01000010) with 99.5% in EzBioCloud database BLAST analysis. Default parameters were used for all software unless otherwise specified. The genome size of JC660 was 2,686,240 bp, with a G + C content of 48.5 mol%. It contained 12 contigs with an *N*_50_ value of 442,168. The annotation by PGAP predicted 2,645 coding sequences, among which 2,623 codes for proteins and 66 genes for RNAs. The genome showed one copy of 5S rRNA and three copies each of 23S rRNA and 16S rRNA genes. The Antismash version 6.0 analysis was done (6) by selecting all the parameters available for the detection of secondary metabolite biosynthesis gene clusters, which showed clusters for terpenes. The metabolic annotation showed that the majority of the genes were mapped to the metabolism of Amino Acids and Derivatives (246), followed by Carbohydrates (147), Cofactors, Vitamins, Prosthetic Groups, Pigments (131), and Membrane transport (610). The *in silico* metabolic annotation of the genome of strain JC660 predicted Mg^2+^-transport-ATPase-associated protein-biofilm-forming genes, genes for organic acid trap transporters, and EcsAB transporters, which are involved in the transport of proteins across membranes (7).

## Data Availability

The whole genome for *Salinicoccus* sp. JC660 has been deposited at GenBank under the accession number, BioProject, and BioSample accession numbers JAJNCU000000000 (https://www.ncbi.nlm.nih.gov/nuccore/JAJNCU000000000), PRJNA782371 (https://www.ncbi.nlm.nih.gov/bioproject/PRJNA782371), and SAMN23377871 (https://www.ncbi.nlm.nih.gov/biosample/?term=SAMN23377871) and SRR17041553 (https://www.ncbi.nlm.nih.gov/sra/?term=SRR17041553), respectively. *Salinicoccus* sp. JC660 is available as NBRC114180 and MCC 4232.

## References

[1] Ramana CV, Srinivas A, Subhash Y, Tushar L, Mukherjee T, Kiran PU, Sasikala C. 2013. Salinicoccus halitifaciens sp. nov., a novel bacterium participating in halite formation. Antonie Van Leeuwenhoek 103:885–898. doi:10.1007/s10482-012-9870-4 23307136

[2] Hyun DW, Whon TW, Cho YJ, Chun J, Kim MS, Jung MJ, Shin NR, Kim JY, Kim PS, Yun JH, Lee J, Oh SJ, Bae JW. 2013. Genome sequence of the moderately halophilic bacterium Salinicoccus carnicancri type strain Crm^T^ (= DSM 23852^T^). Stand Genomic Sci 8:255–263. doi:10.4056/sigs.3967649 23991257 PMC3746416

[3] Bolger AM, Lohse M, Usadel B. 2014. Trimmomatic: a flexible trimmer for Illumina sequence data. Bioinformatics 30:2114–2120. doi:10.1093/bioinformatics/btu170 24695404 PMC4103590

[4] Li R, Zhu H, Ruan J, Qian W, Fang X, Shi Z, Li Y, Li S, Shan G, Kristiansen K, Li S, Yang H, Wang J, Wang J. 2010. De novo assembly of human genomes with massively parallel short read sequencing. Genome Res 20:265–272. doi:10.1101/gr.097261.109 20019144 PMC2813482

[5] Tatusova T, DiCuccio M, Badretdin A, Chetvernin V, Nawrocki EP, Zaslavsky L, Lomsadze A, Pruitt KD, Borodovsky M, Ostell J. 2016. NCBI prokaryotic genome annotation pipeline. Nucleic Acids Res 44:6614–6624. doi:10.1093/nar/gkw569 27342282 PMC5001611

[6] Blin K, Shaw S, Kloosterman AM, Charlop-Powers Z, van Wezel GP, Medema MH, Weber T. 2021. antiSMASH 6.0: improving cluster detection and comparison capabilities. Nucleic Acids Res 49:W29–W35. doi:10.1093/nar/gkab335 33978755 PMC8262755

[7] McLean R, Brown E. 2020. Potential influences of bacterial cell surfaces and nano-sized cell fragments on struvite biomineralization. Crystals (Basel) 10:706. doi:10.3390/cryst10080706

